# A Novel Procedure of Enhancing the Throughput of a Mode-2 Sidelink Based on Partial Sensing

**DOI:** 10.3390/s21186128

**Published:** 2021-09-13

**Authors:** Seokwon Kang, Seungwon Choi

**Affiliations:** Department of Electronic Engineering, Hanyang University, Wangsimni-ro 2222, Seongdong-gu, Seoul 04763, Korea; seokwon.kang@dsplab.hanyang.ac.kr

**Keywords:** 5G NR mode-2 sidelink, V2X, partial sensing, roadside unit

## Abstract

Partial sensing is used to reduce the power consumption of pedestrian user equipment (P-UE) that operates in the signal environment of a mode-2 sidelink. However, because the data trans-mission is allowed only for the window duration of each corresponding P-UE, the throughput of the P-UE decreases by the ratio between the width of the window and the entire data period. This paper presents a novel method for enhancing the throughput of the P-UE that operates with partial sensing in the mode-2 sidelink. The proposed technique employs an additional UE, denoted the roadside unit (RSU), to collect the sensing results from each P-UE that operates with partial sensing. The proposed RSU sequentially aligns all of the partial sensing windows, such that the combination of each partial sensing window can eventually provide an almost complete sensing result. In this study, extensive computer simulations were performed. The results reveal that the proposed method enhances the throughput of each P-UE operating with partial sensing almost to that of full sensing without increasing the required power consumption.

## 1. Introduction

In the 3rd Generation Partnership Project (3GPP), extensive ongoing discussions and standardizations are underway regarding the fifth-generation new radio (5G NR) [1]. Recently, as the interest in autonomous vehicles has increased, discussions regarding 5G NR vehicular communication have actively increased [2]. Latency is one of the most important issues in 5G NR vehicle-to-everything (V2X) communication [3]. Specifically, because safety-related traffic requires low latency, uplink and downlink communications that pass through a base station may not meet the requirements for such a short delay. To support such a low latency, some studies have used a method of direct communication between terminals, termed sidelink communication [4]. Two different categories of side-link communication are known based on the resource allocation method: mode-1 communication and mode-2 communication.

Mode-1 communication is a method wherein a base station allocates usable resources for direct communication between terminals and can be used only when all terminals that perform sidelink communication are in an in-coverage situation.

Mode-2 communication is a method wherein each terminal selects usable resources for direct communication. Mode-2 communication can be used even when the terminals are in an out-of-coverage situation. However, because the base station does not intervene in resource allocation, the terminal needs to identify usable resources itself, and the base station is required to implement a sensing process to determine which resources each terminal can use.

Sensing is used for identifying resources that can be used for the sidelink, in order to decode the physical sidelink control channel (PSCCH) during a sensing window of a certain period before performing the sidelink transmission. However, sensing consumes a large amount of power because the PSCCH needs to be continuously decoded. According to [5], in the case of unicast communication, sensing accounts for 88.7% of the total power consumption, and, in the case of groupcast communication, 35.1% of the total power is used for sensing. In the case of vehicular user equipment (V-UE), power consumption is not a major problem because the power is provided by the vehicle; however, in the case of pedestrian user equipment (P-UE), the power consumption for sensing should be reduced because the battery life of each P-UE is extremely critical. To address these power consumption issues for sensing, a technology called partial sensing has been introduced for sidelink communication among P-UEs [6].

Partial sensing is a method of checking available resources by decoding PSCCH for only a part of the entire data period. When partial sensing is used, the power consumption is reduced as much as the decoding time is reduced. Although the power consumption can be considerably reduced using partial sensing, the throughput must be reduced because the validity of the usable resources is limited only during the period corresponding to the partial sensing window. Consequently, the throughput cannot be reduced as much as the amount of power saved, which is equivalent to the ratio of the partial sensing window duration to the entire data period.

Although a number of pioneering works [7,8,9,10] have addressed the scheduling techniques for resource allocation, most efforts in these studies have been focused on preserving the Basic Safety Message (BSM) [11] by reducing the collision rate among mode-2 sidelink devices. More specifically, [7] presented an approach to avoid the collision of safety messages using Lookahead Information within the sidelink control information, whereas [8] provided an accurate resource allocation state via a Foreseeing Semi-Persistent scheme. Furthermore, [9] considered a special condition in which each mobile device uses its own resource reservation interval to avoid the collision without introducing additional control information, whereas [10] noted the problem of collision between the Random Selection Device (RSD) and the sensing device using the information provided from the RSD.

Sidelink, however, can be employed for the transmission of various kinds of multimedia data, in addition to the BSM only [12]. Furthermore, even when data transmissions among mobile devices cannot be properly handled through a given base station due to the congestion of the mobile devices in a particular area, the sidelink can be adopted to relay the data directly among the mobile devices [13]. Considering these kinds of sidelink scenarios, there is a clear need for a novel method of enhancing the throughput of all the mobile devices involved in a given sidelink scenario without sacrificing the merit of partial sensing, i.e., power saving in resource allocation.

The key contribution of this study is that it proposes a novel procedure for the enhancement of the throughput of each P-UE that participates in mode-2 sidelink communication with partial sensing. The proposed technique employs additional UE, denoted the roadside unit (RSU), which aligns the partial sensing window of each P-UE to combine all of the aligned sensing results. Subsequently, the combined sensing results are transmitted from the RSU back to each P-UE to be shared by all the P-UEs. Based on the proposed technique, the throughput of each P-UE can be increased almost to that achieved in full sensing, while the merit of power saving is still maintained, as in the case of partial sensing.

The remainder of this paper is organized as follows. Section 2 introduces sensing-based mode-2 communication. Section 3 introduces the procedure to combine all the partial sensing results of the method presented in this paper. Section 4 presents the simulation results, and Section 5 concludes the paper.

## 2. Sensing-Based Mode-2 Sidelink Communication

In the 5G NR mode-2 sidelink, the base station does not intervene in the resource selection process of the terminal, meaning that each terminal selects the usable resources itself. Each terminal performs the sensing procedure by decoding the PSCCH to determine which resources are not occupied by other terminals. Therefore, sensing is performed prior to data transmission. In this section, we summarize the basic philosophy of the conventional resource allocation procedures based on resource sensing.

**Figure 1:**
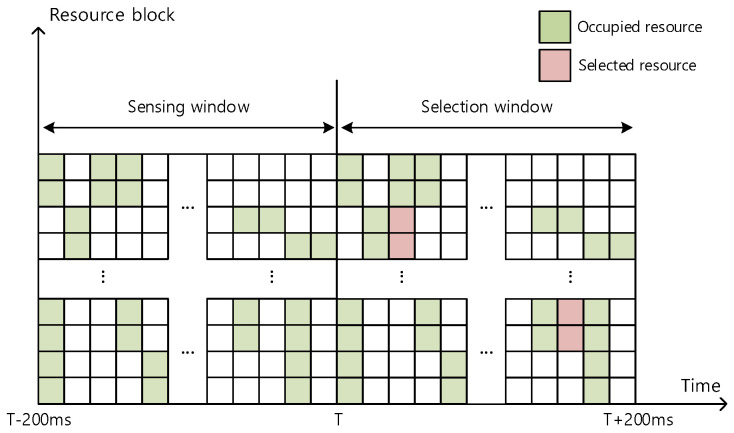
Sensing window and selection window.

Figure 1 presents a timing diagram of the sensing and selection process when the length of the sensing window is set to 200 ms. This corresponds to the method of full sensing which can be applied to V-UEs, for which considerably less severe restrictions on power consumption are imposed. In Figure 1, T denotes the timing corresponding to the start of the selection window. The sensing window starts at time T − 200 ms, that is, 200 ms before T. During the sensing window period, each terminal undergoes the sensing process of decoding the PSCCH resource blocks used by all terminals. Thus, sensing is used to identify the occupied resources and exclude the occupied resource blocks from the candidate resource set. After the candidate resource set is obtained through this process, the resource blocks to be used for data transmission are selected from the candidate resource set that is used during the selection window. Through this process, even if the base station does not allocate resource blocks, the terminal can identify the resource blocks being used by all other terminals and thus prevent a collision.

Random selection is a method in which usable resources are randomly selected and data are transmitted without sensing the resource blocks occupied by other terminals. Although no power is consumed for sensing, high collision probability can be achieved because each terminal transmits data without sensing.

**Figure 2:**
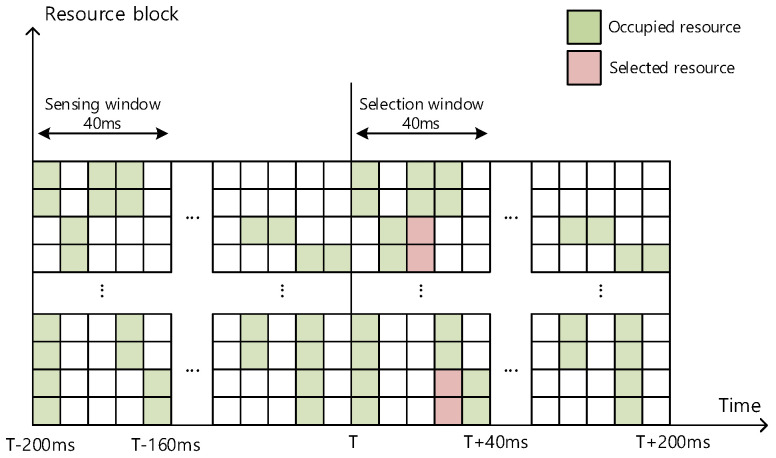
Partial sensing window and selection window.

Figure 2 presents a timing diagram for the method of partial sensing considered mainly for P-UEs, which suffer significant restrictions on power consumption, where sensing is performed only for a partial sensing window of 40 ms for the entire observation period of 200 ms. As shown in Figure 1, T in Figure 2 also denotes the timing where the selection window begins. Here, sensing at each terminal is performed only for 40 ms, corresponding to the time interval from T − 200 to T − 160 ms. Because PSCCH decoding is performed only for 40 ms (from T − 200 to T − 160 ms), the selection window is eventually reduced to 40 ms (from T to T + 40 ms). Consequently, usable resources are selected only for the selection window of 40 ms.

With partial sensing, power consumption can be reduced to approximately 1/5 (=40 ms/200 ms) compared to the case of sensing the resources for the entire 200 ms observation period. It can also prevent collisions, unlike in random selection. However, because sensing is performed only for a 40 ms partial window, selectable resources are also limited only for the corresponding time interval. Consequently, the throughput is reduced to nearly 1/5 (=40 ms/200 ms). We claim that, if several P-UEs can share their sensing results with each other, the throughput may be substantially improved. In the next section, we present a method for combining all the partial sensing results.

## 3. How to Combine All the Partial Sensing Results

To describe the novel procedure of combining all the partial sensing results, we first introduce an additional UE, denoted RSU, in the region of mode-2 sidelink communications of interest. The RSU is a hardware entity that is generally defined as a terminal to assist vehicular communications in out-of-coverage areas [14].

**Figure 3:**
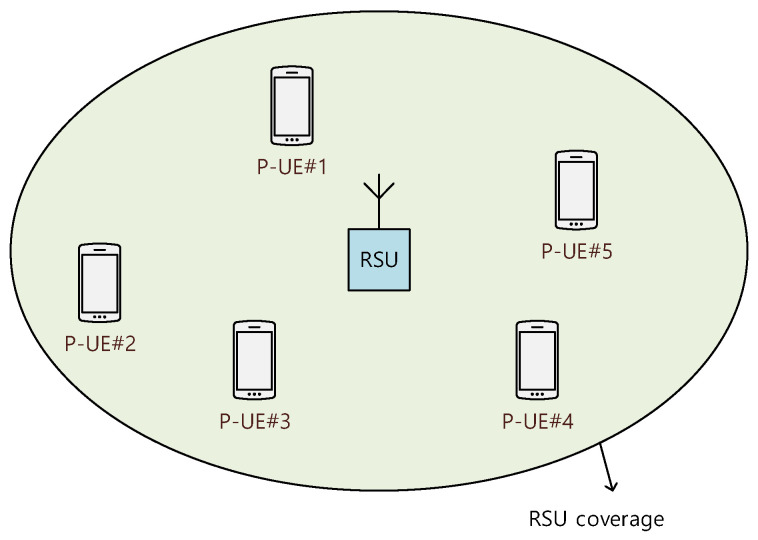
A mode-2 sidelink signal environment consisting of an RSU and P-UEs.

Figure 3 illustrates a mode-2 sidelink signal environment, including several P-UEs within the coverage of an RSU.

**Figure 4:**
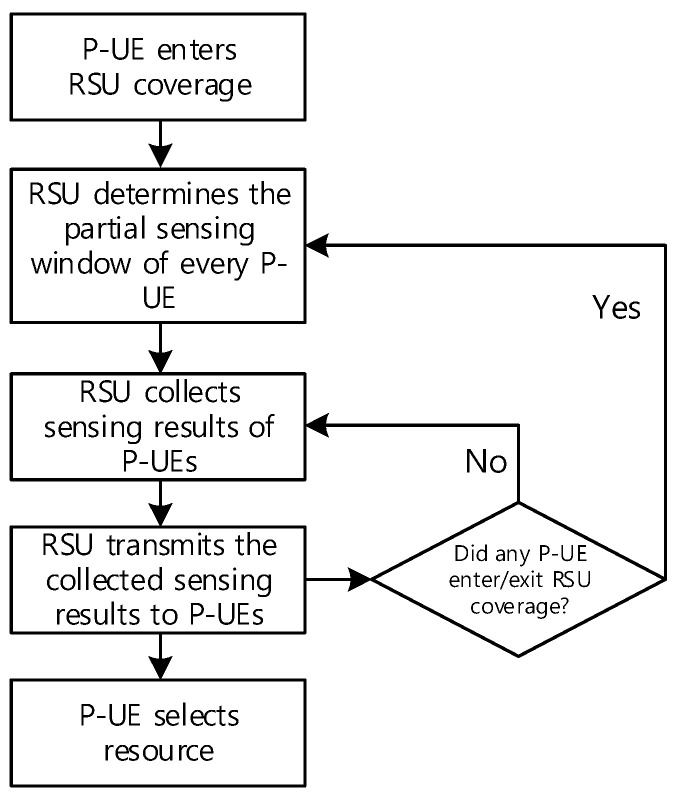
Proposed method of combining all the partial sensing results using the RSU.

Each P-UE operates with partial sensing to perform mode-2 sidelink communication with minimal power consumption. We propose a novel procedure that combines all the partial sensing results under this signal environment, as shown in Figure 4.

First, the RSU assigns a partial sensing window to each P-UE whenever a new P-UE (or new P-UEs) enters or exits its coverage area. When assigning the partial sensing window, it is important to ensure that each partial sensing window does not overlap among different P-UEs for efficient partial sensing.

Second, the RSU collects all the partial sensing results from all P-UEs. As each P-UE decodes the sidelink control information from its PSCCH, the P-UE can determine how the resource block of its PSSCH is occupied. The resource block occupation is then transmitted from each P-UE to the RSU such that the state of the resource block occupation of all the P-UEs within the RSU coverage can be collected.

Third, the RSU transmits the collected information to all the P-UEs. Consequently, each P-UE becomes aware of all the sensing results for the other time slots, in addition to its own time slot.

Finally, to check whether any P-UE enters or exits the RSU coverage, the RSU realigns all the partial sensing windows upon any changes within its coverage.

**Figure 5:**
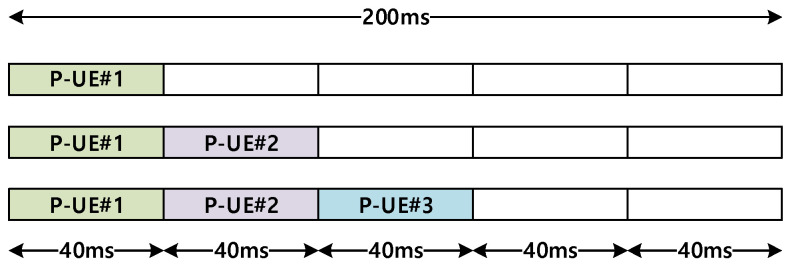
Aligning of partial sensing window for P-UEs.

Figure 5 illustrates how the partial sensing window is aligned for each P-UE within the entire period of reserving its resource, that is, 200 ms, with a window duration of 40 ms [15]. Note that each partial sensing window is assigned to a different P-UE, meaning that each window is assigned to a distinct P-UE. However, if the total number of P-UEs within the RSU coverage exceeds five, overlap is unavoidable.

As shown in Figure 5, the first P-UE is assigned to the first window of 0–40 ms by the RSU. Similarly, the second and third P-UEs are assigned to the second and third windows, that is, for 40–80 ms and 80–120 ms, respectively. By aligning the partial sensing windows in this manner, the RSU can most efficiently acquire the state of resource occupation of all the P-UEs by summing the sensing results corresponding to all the different time slots. As the RSU transmits the combined sensing information to each P-UE, each P-UE becomes aware of the resource occupation state for the entire observation period of 200 ms, which is typically available only in the case of full sensing.

**Figure 6:**
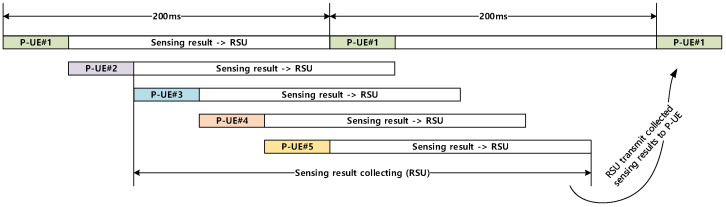
Timing diagram for exchanging the sensing results between RSU and P-UEs.

Figure 6 illustrates a timing diagram for exchanging the sensing results between the RSU and each P-UE, which is briefly described in the second and third steps of Figure 4. Each P-UE sends its sensing result to the RSU during the current 200 ms period. As shown in Figure 6, the RSU collects all the sensing results from the corresponding five P-UEs before the end of the next observation period. When the combined sensing results are received from the RSU, each P-UE is ready to select an appropriate set of resources from the beginning of the second observation period, which is 400 ms away, because of the commencement of the partial sensing of the first P-UE (P-UE#1).

**Figure 7:**
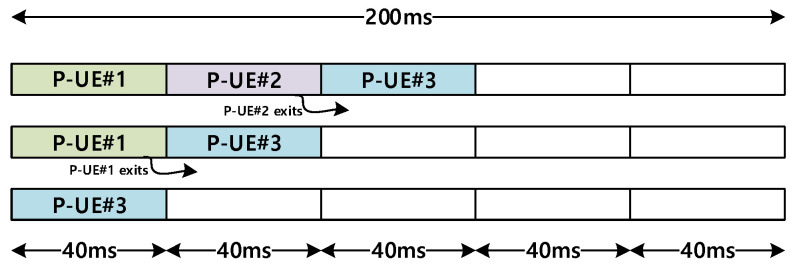
Realigning of partial sensing windows for P-UEs exiting the RSU coverage.

Figure 7 illustrates how the partial sensing windows should be realigned as a P-UE (or P-UEs) exits the RSU coverage. P-UE#2 and P-UE#1 exit the RSU coverage from an initial state wherein the three P-UEs have been aligned, as shown at the top in Figure 7. As P-UE#2, whose partial sensing window has a time slot of 40–80 ms, exits the coverage, the partial sensing window of P-UE#3 occupies this time slot. Similarly, as P-UE#1, which occupied 0–40 ms, exits, P-UE#3 takes this time slot for partial sensing.

Through the abovementioned procedure, each P-UE can acquire the sensing results for the time slot corresponding to its own window duration of 40 ms, and for the entire observation period of 200 ms. The only possible conflict included in the proposed procedure is that, as briefly mentioned in Figure 6, each P-UE selects its resources based on the sensing results obtained approximately 400 ms before the actual transmission. The key part of the proposed technique is that the total throughput of each P-UE is expected to increase almost to that of full sensing, which is five-fold larger than that of the conventional partial sensing method.

## 4. Simulation Results

All the numerical results presented in this section were obtained using the sidelink functions of Release 14 (Rel-14) Long Term Evolution (LTE) provided by the MATLAB LTE Toolbox [16]. Although the proposed technique mainly targets the 5G NR, all the computer simulations in this study were performed for the signal environment of the Rel-14 LTE rather than the 5G NR, because the specification for the 5G NR sidelink has not yet been released. Thus, the performance of the proposed method, which employs the RSU as a collector of the partial sensing results, is compared to that of the conventional partial sensing in the signal environment of Rel-14 LTE. However, the proposed method introduced in Section 3 can be applied to the 5G NR with no changes, except that new parameter values for the 5G NR, for example, data period and/or window width, may need to be adopted.

In our simulations, the data period and partial sensing duration were set to 200 and 40 ms, respectively, as specified in the Rel-14 LTE. The resource pool for the partial-sensing-based sidelink comprises subcarriers with a width of 5 MHz and a PSCCH period of 40 ms. In the case of time-division duplexing, the 40 ms PSCCH period, comprising 40 subframes, includes both the reception and transmission parts, each of which is filled with two subframes of PSCCH and 16 subframes of the physical sidelink shared channel, whereas the remaining four subframes are empty. These parameter values can be modified based on 5G NR specifications to apply the proposed technique to the 5G NR.

To simulate a practical communication environment of the mode-2 sidelink, the collective perception of the environment (CPE), one of the sidelink use cases, was assumed [17]. In the assumed CPE, several P-UEs and V-UEs communicate 1600 bytes of CPE traffic with one another every 200 ms. Based on the philosophy of the partial sensing technology, that is, to reduce the battery power consumption, each P-UE checks the traffic resources occupied by all the V-UEs and the other P-UEs only for the 40 ms of the partial sensing window, instead of the entire observation period of 200 ms. Subsequently, each P-UE determines the resources that are available for use during its transmission period.

All computer simulations presented in this section were performed in an urban signal environment of the mode-2 sidelink, as shown in Figure 3. The transmit power of the RSU and P-UE was 28 and 22 dBm, respectively; thus, the RSU coverage and inter-P-UE coverage were 100 and 50 m, respectively [18,19].

**Figure 8:**
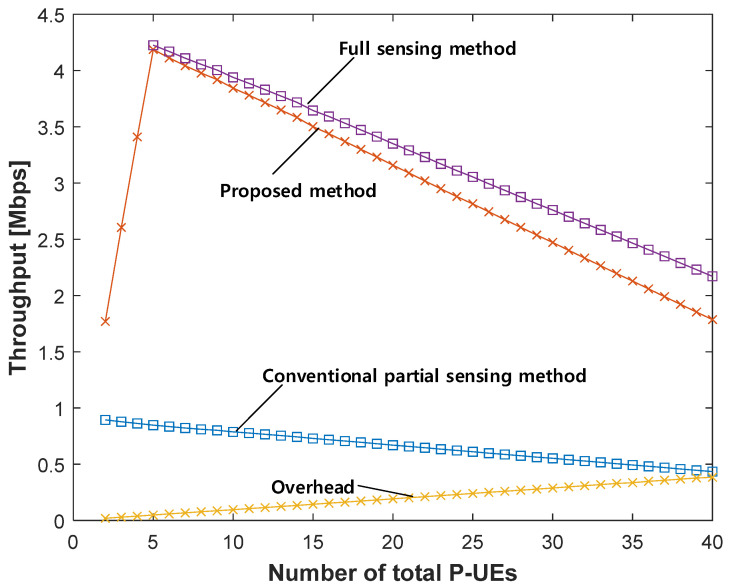
Throughput comparison and required overhead.

Figure 8 compares the maximum possible throughput of each P-UE obtained by the proposed method to that of the conventional partial sensing method. As a reference, the throughput provided by the full sensing method is also shown. In addition, the overhead required for the proposed method to exchange the sensing information between the RSU and each P-UE is also presented.

To analyze the results shown in Figure 8, we first compute the number of P-UEs that can be served by the proposed method with a given resource pool. As shown above in Figure 4, to ensure that the proposed method provides the combined partial sensing results to each P-UE, each P-UE should first transmit its sensing result to the RSU (consuming two PSCCH resource blocks). Subsequently, the RSU re-transmits the combined sensing results to each P-UE (consuming additional two PSCCH resource blocks). Finally, each P-UE can transmit its traffic (consuming additional two PSCCH resource blocks) [20]. Because each of the three steps consumes two PSCCH resource blocks, each P-UE needs six PSCCH resource blocks based on the procedure of the proposed method. Because 50 PSCCH resource blocks exist within the resource pool of 5 MHz bandwidth, the maximum number of P-UEs per 40 ms partial sensing window is 8 (= 50/6); thus, at most 40 (= 8 × 5) P-UEs can exist for the entire observation period of 200 ms.

Figure 8 illustrates the values for throughputs provided to each of the P-UEs by the full sensing method, conventional partial sensing method, and proposed method, together with the overhead incurred in the proposed method. In this figure, it can be observed that, as the number of P-UEs increases from two to five, the maximum throughput that can be provided to each P-UE increases proportionally with the number of P-UEs in the proposed method. Importantly, each P-UE can exploit the sensing results of the other P-UE and its sensing result without overlapping, as long as the number of P-UEs does not exceed five. In contrast, the throughput of the conventional partial sensing method decreases as the number of P-UEs increases. The major reason for this is that the CPE traffic must increase as the number of P-UEs increases. Although an increase in CPE traffic occurs in the case of the proposed method, the simulation results show that the effect of the CPE traffic increase is almost negligible compared to the benefit of providing the combined sensing results. Furthermore, the throughput of each P-UE starts decreasing as the number of P-UEs exceeds five. The main reason for this is that, when the number of P-UEs exceeds five, the additional benefit of combining the partial sensing results obtained from more than five P-UEs is negligible because the number of partial sensing windows that can be combined is fixed to five; thus, the combination of the partial sensing results from more than five P-UEs is no longer helpful. Moreover, the overhead required to exchange the sensing results between the RSU and each of the P-UEs, and the CPE traffic, increases as the number of P-UEs further increases. Regarding the overhead shown in Figure 8, because 1 PSSCH resource block is needed for transmitting the sensing result from the P-UE to RSU, and three resource blocks are needed for transmitting the results back from RSU to each P-UE, the total overhead can be obtained easily from the formula given in [15].

The throughput provided by the full sensing method was approximately five-fold that of the conventional partial sensing method. This is because, as mentioned earlier, usable resources can be selected during the entire 200 ms observation period, which is five times longer than the 40 ms partial sensing window. The slight inferiority of the proposed method compared to full sensing mainly results from the overhead required for the proposed method to exchange the partial sensing results between the RSU and each of the P-UEs. In terms of the benefit of reducing the power consumption provided by the proposed method, however, the slight difference in the achievable throughput between the full sensing and proposed methods is negligible. In practice, the importance of the battery life of each P-UE cannot be overemphasized.

As shown in Figure 8, when the number of P-UEs is five, the throughput of each P-UE provided by the proposed method (4.187 Mbps) is approximately five-fold that of the conventional partial sensing method (0.847 Mbps). The superiority of the proposed method remains consistent even when the number of P-UEs increases up to 40, with results of 1.786 Mbps (proposed) versus 0.434 Mbps (conventional).

We present the application of the proposed method of combining the partial sensing results to the case when the total number of P-UEs exceeds 40 during the observation period of 200 ms. In the proposed method described in Section 3, all the P-UEs transmit the RSU partial sensing results such that the RSU re-transmits the combined sensing results to each P-UE. Generally, the partial sensing result provided by every P-UE within a given 40 ms time slot would be almost the same because each partial sensing procedure is performed within a given RSU coverage. That is, when more than one P-UE is assigned for partial sensing within a single time slot, the partial sensing results reported to the RSU by all the P-UEs must overlap with one another. The partial sensing result of one P-UE would be different from that of another only when the P-UEs are located far away from each other within the RSU coverage, such that they afford distinct partial sensing results. Based on this analysis, every P-UE in each 40 ms time slot need not send the partial sensing result to the RSU. In an extreme case, only one P-UE in each 40 ms time slot is assigned to report its partial sensing result to the RSU, whereas all the other P-UEs in the corresponding 40 ms time slot do not transmit their partial sensing results to the RSU such that they do not occupy the corresponding resources of the two PSCCH resource blocks. Consequently, the maximum number of P-UEs per 40 ms partial sensing window can be increased to 11, meaning that up to 55 P-UEs can exist for the entire observation period of 200 ms. With this modification of the proposed method, the overhead required for exchanging the partial sensing results between the RSU and each P-UE is also considerably reduced.

Table 1 shows the number of P-UEs that can be served by the proposed method according to the number of P-UEs reporting their partial sensing results to the RSU.

**Figure 9:**
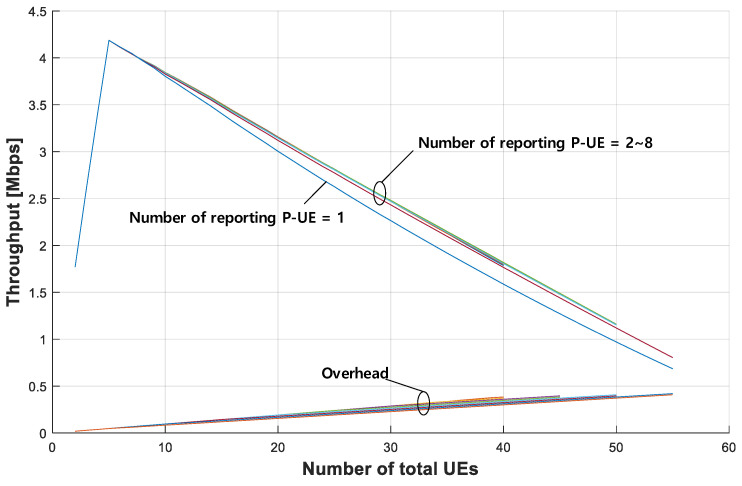
Throughput and overhead of the modified proposed method.

As the number of reporting P-UEs decreases, although the overhead also decreases, the maximum throughput of each P-UE remains almost the same as that in Figure 8, where all the P-UEs in each 40 ms time slot report their partial sensing results to the RSU. Although the collision probability must increase as the number of reporting P-UEs decreases, as shown in Figure 9, the impact of reducing the number of P-UEs is not negligible unless this number is reduced to one. This result is consistent with that of the earlier analysis regarding the overlap among the partial sensing results reported by multiple P-UEs in a given 40 ms time slot.

**Figure 10:**
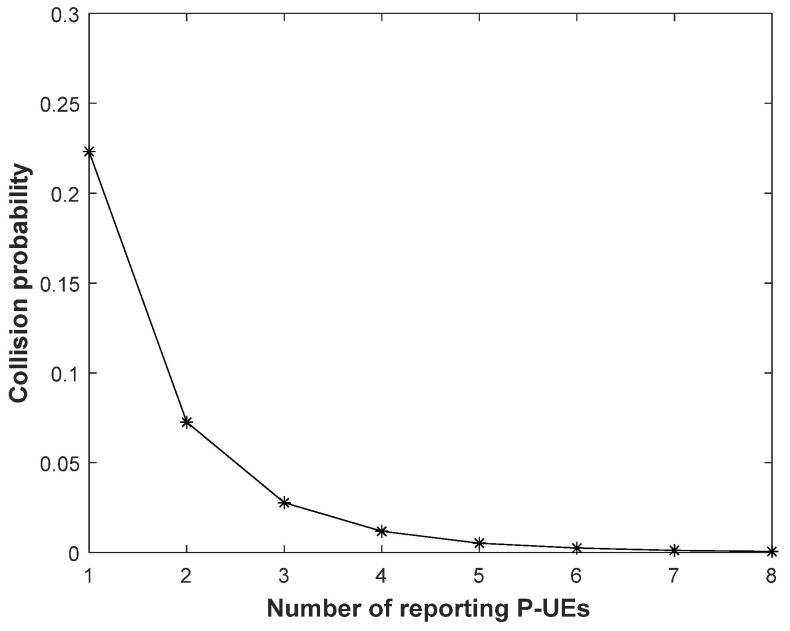
Collision probability.

Figure 10 illustrates the collision probability as the number of reporting P-UEs varies from one to eight. It is worth noting that the collision probability was computed under the assumption that the 200 ms observation period is occupied by the maximum supportable number of P-UEs for each case, that is, 55, 55, 50, 50, 45, 45, 40, or 40 P-UEs are to be served, where the number of reporting P-UEs at each 40 ms time slot is 1, 2, 3, 4, 5, 6, 7, or 8, respectively, as shown in Table 1. As shown in Figure 10, the collision probability rapidly decreases as the number of reported P-UEs exceeds one. When only one P-UE reports its partial sensing result to the RSU, the collision probability in the signal environment considered in this study was found to be 22.5%. The collision probability is significantly reduced as the number of reporting P-UEs exceeds one; therefore, the throughput performance shown in Figure 9 is comparable to that of Figure 8 unless the number of reporting P-UEs is one. Consequently, the number of reporting P-UEs, which should not be one, as discussed above, does not exceed two in the signal environment considered in this study; thus, the total number of supportable P-UEs can be increased to 55, as shown in Table 1.

## 5. Conclusions

The proposed method of combining the partial sensing results obtained from each P-UE provides a throughput comparable to that of full sensing while maintaining the merit of power saving offered by partial sensing. From the extensive simulations performed in a mode-2 sidelink environment, that is, CPE, it was observed that the proposed method increased the maximum throughput of each P-UE 4.94-fold when there were five P-UEs compared to that in the conventional partial sensing method. When the total number of P-UEs was 40, the throughput increased 4.11-fold due to the increase in the overhead and resource occupancy.

The maximum number of P-UEs that can be supported by the proposed method was limited to 40 because of the quantity of PSCCH resources required for each of the reporting P-UEs. Thus, the proposed method was modified such that only some P-UEs (rather than all of them) for each time slot reported their partial sensing results to the RSU. Consequently, the maximum number of P-UEs that can be served by the proposed method can be increased in accordance with the reduced number of reported P-UEs. When the number of reporting P-UEs decreases to one, which allows for the maximum number of P-UEs to increase to 55, the throughput performance is considerably inferior to the case in which the number of reporting P-UEs exceeds one. This is because the collision probability is high when only a single P-UE reports its partial sensing result to the RSU. As the number of reporting P-UEs exceeds one, the collision probability decreases rapidly, resulting in a throughput comparable to the case of employing all eight P-UEs as reporters. Consequently, based on the simulation results, it appears to be most suitable to use two reporting P-UEs. The simulation result regarding the required number of reporting P-UEs to fully exploit the merit of the proposed method may change depending on various environmental parameters, such as the transmit power, and the coverage of the RSU and each P-UE, which were set to 28 and 22 dBm, and 100 and 50 m, respectively.

All of the approaches discussed in this paper were implemented under the assumption that there exists a single RSU among a number of P-UEs whose resources are to be properly allocated without collision. If there exists a number of RSUs in a given sidelink, meaning that there are multiple sets of P-UEs, each including a number of P-UEs sharing their sensing results via their own RSU, the sensing results of every RSU should also be shared. This scenario of multiple RSUs is left for future research. The realization of this scenario may yield results with greater practical value while imposing extra complexity and latency.

## Figures and Tables

**Table 1:** The number of P-UEs that can be served by the proposed method according to the number of P-UEs reporting their partial sensing results to the RSU.

Number of reporting P-UEs/40 ms partial sensing window	1	2	3	4	5	6	7	8
Number of total P-UEs/200 ms observation period	55	55	50	50	45	45	40	40

## Data Availability

Not applicable.
